# Diagnosis and Treatment of Vulvo-Perineal Endometriosis: A Systematic Review

**DOI:** 10.3389/fsurg.2021.637180

**Published:** 2021-05-11

**Authors:** Charlotte Maillard, Zineb Cherif Alami, Jean-Luc Squifflet, Mathieu Luyckx, Pascale Jadoul, Viju Thomas, Christine Wyns

**Affiliations:** ^1^Department of Gynecology-Andrology, Cliniques Universitaires Saint-Luc, Université Catholique de Louvain, Brussels, Belgium; ^2^Department of Obstetrics and Gynecology, Clinique Saint-Jean, Brussels, Belgium; ^3^Tumor Infiltrating Lymphocytes Group - De Duve Institute, Université Catholique de Louvain, Brussels, Belgium; ^4^Department of Obstetrics and Gynecology, Tygerberg Hospital, University of Stellenbosch, Cape Town, South Africa; ^5^Institut de Recherche Expérimentale et Clinique, Université catholique de Louvain, Brussels, Belgium

**Keywords:** endometriosis, perineum, vulva, episiotomy, perineal pain, cyclical pain, perineal nodule, extrapelvic endometriosis

## Abstract

**Objective:** To describe the available knowledge on vulvo-perineal endometriosis including its diagnosis, clinical management and recurrence rate.

**Methods:** We followed the PRISMA guidelines for Systematic Reviews and our study was prospectively registered with PROSPERO (CRD42020202441). The terms “*Endometriosis”* and “*Perineum”* or “*Vulva”* were used as keywords. Cochrane Library, Medline/Pubmed, Embase and Clinicaltrials.gov were searched. Papers in English, Spanish, Portuguese, French or Italian from inception to July 30, 2020 were considered. Reference lists of included articles and other literature source such as Google Scholar were also manually scrutinized in order to identify other relevant studies. Two independent reviewers screened potentially eligible studies according to inclusion criteria.

**Results:** Out of 539 reports, 90 studies were eligible including a total of 283 patients. Their mean age was 32.7 ± 7.6 years. Two hundred sixty-three (95.3%) presenting with vulvo-perineal endometriosis have undergone either episiotomy, perineal trauma or vaginal injury or surgery. Only 13 patients (4.7%) developed vulvo-vaginal endometriosis spontaneously i.e., without any apparent condition favoring it. The reasons that motivated the patients to take medical advice were vulvo-perineal cyclical pain increasing during menstruations (98.2% of the patients, *n* = 278). Out of the 281 patients for whom a clinical examination was described, 274 patients (97.5%) showed a vulvo-perineal nodule, mass or swelling while six presented with bluish cutaneous lesions (2.1%) and 1 with bilateral polyps of the labia minora (0.4%). All but one patients underwent surgical excision of their lesions but only 88 patients (28.1%) received additional hormonal therapy. The recurrence rate was 10.2% (29 patients) considering a median follow-up period of 10 months (based on 61 studies).

**Conclusion:** In conclusion, vulvo-perineal endometriosis is a rare entity with approximately 300 cases reported in the literature since 1923. With the available knowledge shown in this systematic review, we encourage all practitioners to think about perineal endometriosis in case of perineal cyclical pain with or without previous perineal damage. Diagnosis should be done with clinical exam, perineal ultrasound and pelvic MRI when available. In case of anal sphincter involvement, perianal ultrasound should be performed. Surgical excision of the lesion should be realized in order to remove the lesion and to confirm the diagnosis histologically. Hormonal treatment could be proposed to attempt to decrease the size of a large lesion before surgery or to avoid recurrence of the lesion. As evidence-based approach to the diagnosis, treatment and recurrence rate of affected patients remains a challenge given its low prevalence, the variations in management found in the articles included and the limited quality of available studies, we suggest that a prospective database on vulvo-perineal endometriosis should be generated to increase knowledge but also awareness among healthcare professionals and optimize patients' care.

**Systematic Review Registration:** https://www.crd.york.ac.uk/prospero/, identifier: CRD42020202441.

## Introduction

Endometriosis is a complex benign disease characterized by an estrogen-dependent chronic inflammatory process and is defined as the presence of endometrial glands and stroma-like tissue outside the uterine cavity, most often located in the pelvis (1), although extrapelvic sites have been described, including the urinary and gastro-intestinal tracts, the nervous system, the thoracic cavity and diaphragm as well as some (sub-)cutaneous sites (2–5). It occurs in ~10% of women of reproductive-age and in 35–50% of women with infertility and chronic pelvic pain (6–8).

While extrapelvic endometriosis is a relatively uncommon condition, accounting for ~12% of all cases of endometriosis (9), perineal endometriosis is an even rarer entity. Cutaneous and subcutaneous endometriotic lesions have been observed in surgical scars following laparoscopy, laparotomy, vulvo-vaginal surgery, episiotomy and obstetrical lacerations whether surgically repaired or not (3). Perineal endometriosis may involve the skin and/or subcutaneous tissue of the vulva and perineum but also the perianal sphincteric muscular tissue (10). Following the first case of perineal endometriosis reported in 1923 (11), most of what is known of this commonly misdiagnosed entity comes from case reports published over the past 60–70 years.

Our study aims to identify all reported cases of vulvo-perineal endometriosis published in the literature in order to describe diagnostic processes, treatments and recurrence rates of this uncommon type of endometriosis and help practitioners to achieve prompt diagnosis and optimize patients' outcomes.

## Methods

This study followed the principles of the Preferred Reporting Items for Systematic Reviews and Meta-Analysis (PRISMA) statement (12) and was prospectively registered within the International Prospective Register of Systematic Review (PROSPERO) on the 4th of August 2020 (number CRD42020202441).

The following search engines and electronic databases were searched on July 30, 2020 by one author (CM): Cochrane Library, Medline/Pubmed, Embase and Clinicaltrials.gov. The terms “*Endometriosis”* and “*Perineum”* or “*Vulva”* were used as keywords to recover all possible publications. Table 1 shows the queries and record numbers for the different databases used. No restrictions regarding language, type or date of publication were initially applied. Reference lists of included articles and other literature sources such as Google Scholar were also manually scrutinized in order to identify additional relevant studies. Articles in English, Spanish, Portuguese, French or Italian were considered. Review articles and studies describing exclusively lesions involving the abdominal wall or the inguinal area, or malignant transformation of endometriosis were excluded. Two reviewers (CM, ZCA) independently screened titles and abstracts of the search output to identify potentially eligible studies and cross-examined their results.

**Table 1:** Description of queries in the different databases.

	**Query**	**Results**
Pubmed/Medline	(“Endometriosis”[MeSH Terms] OR “Endometriosis”[All Fields]) AND (“perineum”[MeSH Terms] OR “perineum”[All Fields] OR “vulva” [MeSH Terms] OR “vulva”[All Fields])	171
Embase	(“Endometriosis”/exp OR endometriosis) AND (“perineum”/exp OR perineum OR “vulva”/exp OR vulva)	362
Cochrane Library	(endometriosis):ti,ab,kw AND perineum):ti,ab,kw (Word variations have been searched)	1
	(endometriosis):ti,ab,kw AND vulva):ti,ab,kw (Word variations have been searched)	2
ClinicalTrials.gov	(“Endometriosis” AND “Perineum” OR “vulva”)	3
		539

## Results

**Figure 1:**
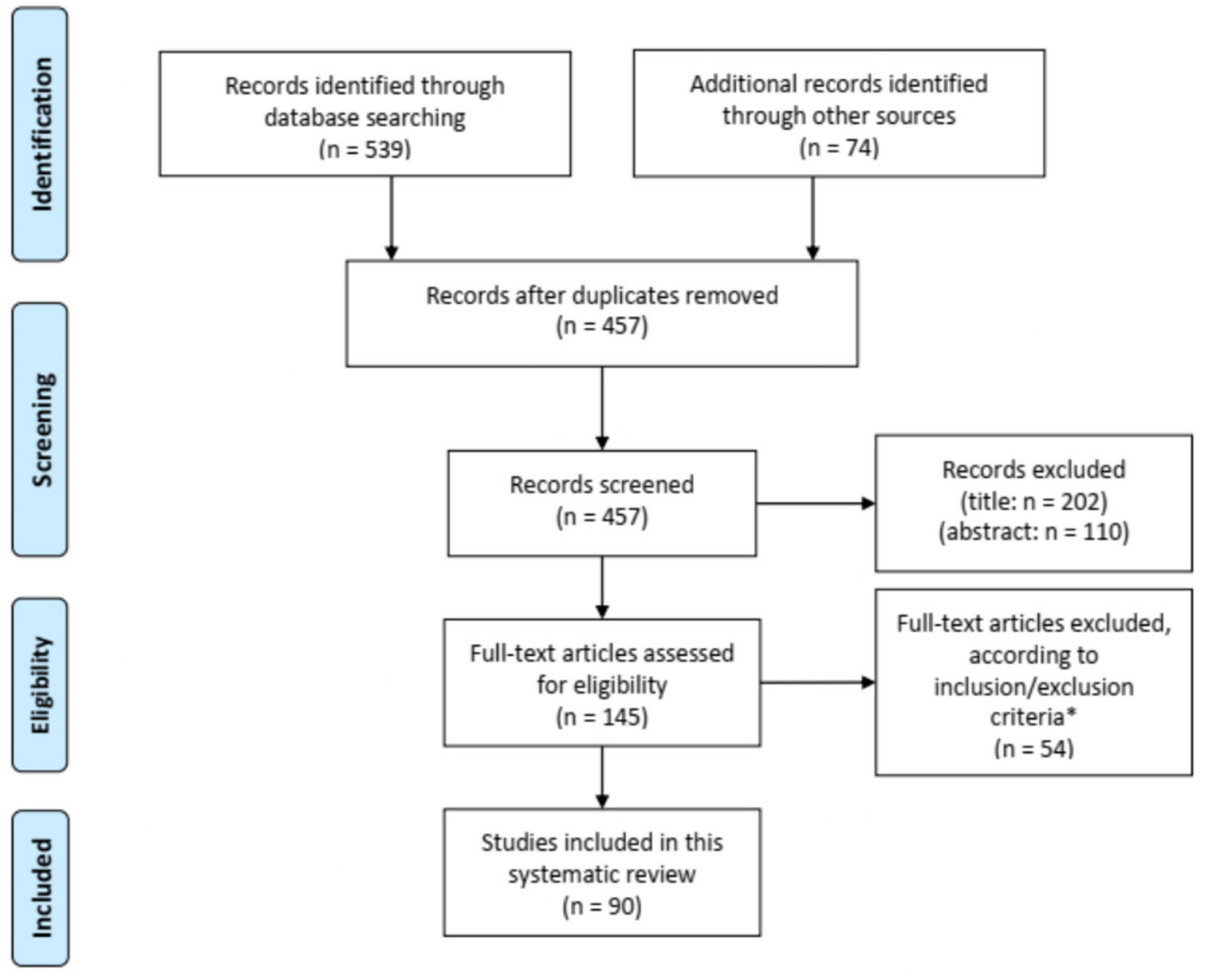
Flow diagram for study selection. *Seven articles could not be retrieved despite contacting the corresponding author.

Figure 1 shows the flow chart of the literature search. Our search strategy yielded a total of 457 potentially eligible studies. Ninety articles published between 1956 and 2020 were eventually included in our review. Eighty-three articles were case reports or case series including one to eight patients (2, 9, 11, 13–90) and seven studies described retrospective cohorts of 14–36 patients (10, 91–96). The main results of the eight retrospectives studies can be found in Table 2.

**Table 2:** Data of the literature about vulvo-perineal endometriosis describing more than 10 cases.

	**Number of cases**	**Mean age (range)**	**Symptoms**			**Vulvo-perineal scar (*n*)**	**IAS**	**Work-up (*n*)**	**Duration of symptoms (range) (months)**	**Latent period (range) (months)**	**Treatment**		**Other endometriosis spots**	**Recurrence**	**Follow-up period (months)**
			**Perineal nodule (%)**	**Progressive and cyclical pain (%)**	**Other (%)**						**Surgical**	**Medical**			
Paull et al. (91)	15	28 (19-34)	100	100	NA	Episiotomy scar (15)	NA	NA	NA	21 (1-60)	Excision	No	NA	0	9: 12–48, 6: NA
Nominato et al. (92)	21	30.8 (1-48)	79	80	NA	Episiotomy scar (19), perineoplasty (2), perineal surgery (1)		NA	NA	44	Excision	No	NA	1	NA
Zhu et al. (93)	36	30.67 (23-44)	100	100	Dysmenorrhea (13.9), dyspareunia (5.6)	Episiotomy (24), perineal tear (12)	26	CA 125 (30), color doppler perineal ultrasound (36)	NA	42.8 (4-156)	Complete excision (28), uncomplete (7), hormonal (1)	GnRHa 3–6 months pre or post surgery (20 with IAS)	3	7 (with incomplete excision) 1 with hormonal treatment alone	0.5–168
Chen et al. (10)	31	33.4 (26-43)	100	100	NA	Episiotomy scar (20), perineal lacerations (11)	31	CA 125, pelvic U/s, perineal U/s (5)	NA	36 (1-204)	Complete narrow excision (30), incomplete excision (1)	NE: 10 pre- and post-op 1–6 months (GnRHa, Nemestran, DMPA), 1 GnRHa 3 months preop-Mirena post-op, 9 GnRH a pre-op 3–5 months, 2 GnRHa 4 months post-op, 8 no treatment, IE: DPMA pre- and post-op 3–6 months	2 (ovarian endometrioma)	2 (NE with GnRHa 3 months, IE)	18 (6-78)
Li et al. (94)	17	34.35 (26-57)	100	100	NA	Episiotomy scar (17)	6	CA 125 (15), doppler U/s (8), pelvic U/s (17), MRI (2)	37.82 (3-152)	46.82 (2-204)	Excision	Post-op: 4 GnRHa (3 months), 2 mifepristone 1 month, 1 progesterone	1	1	74.23 (5-151)
Matalliokis et al. (95)	14	32.5 (±2.9)	92.8	92.8	NA	Episiotomy scar (14)	NA	U/s (3), CT (6), MRI (7)	NA	NA	Excision	NA	3	2	NA
Liu et al. (96)	35	33.44 (25-48)	100	100	NA	Episiotomy scar (33), opposite side of episiotomy (1), mons pubis (1)	10	CA125 (11), perineal U/s (15), pelvic U/s (35)	NA	42.44 (1-120)	Complete excision ± primary sphincteroplasty	preop (7) (5 GnRHa, 1 Marvelon, 1 mifepristone), post op (17) (2 mifepristone, 15 GnRHa)		3 (18.75%) in non GnRHa group, 1 in the gnRHa group	7–86

*U/s, ultrasound; CT-scan, computerized tomography; IAS, involved anal sphincter; CNE, complete narrow excision surgical margin: 0.3–0.5 cm; DMPA, depot pedroxyprogesterone acetate; GnRHa, gonadotropin-releasing hormone agonists; NE, narrow excision*.

Two hundred and eighty-three patients, with a mean age of 32.7 ± 7.6 years and an age range from 14 to 69 years at diagnosis, were included. While 263 patients (95.3%) had undergone previous episiotomy, obstetrical lacerations, whether repaired or not, perineal trauma or vaginal surgery or injury, only 13 patients (4.6%) developed spontaneous vulvo-vaginal endometriosis i.e., in the absence of the most frequently associated conditions (Table 3). Among the latter, lesions developed in the Bartholin gland in 6 cases (21, 44, 48, 62, 70, 80). Previous history wasn't described for seven patients (40, 45, 47, 74).

**Table 3:** Description of previous vulvo-perineal history.

**Vulvo-perineal endometriosis**	**Number of patients (/283)**	**%**
Spontaneous	13	4.6
Previous vulvo-perineal lesion	263	92.9
Episiotomy or obstetrical laceration	249	88
Bartholin cystectomy	4	1.4
Laparotomy (for ovarian endometriosis)	1	0.4
Laparotomy (for ovarian endometriosis) with hernia repair	1	0.4
Laser vulvar surgery	1	0.4
Vaginal hysterectomy	1	0.4
Manchester surgery for prolapse	1	0.4
Mile's procedure for rectal cancer	1	0.4
Vulvar abrasion	1	0.4
Vulvar hematoma post trauma	1	0.4
Vulvar ulceration	1	0.4
Removal of Nuck canal remnant	1	0.4
Not specified	7	2.5

The median latent period i.e., the time between perineal trauma or surgery and occurrence of symptoms was 2.5 years, ranging from 1 month to 14 years.

Incidence rates were reported in two studies, ranging between 0.01 and 0.06% after vaginal deliveries (84, 92). The incidence of anal sphincter involvement was reported only by Chen et al. who described a 0.37% incidence of perineal endometriosis among women treated surgically for endometriosis regardless of its location with 0.18% of patients presenting with anal sphincter involvement (10).

Main complaints were vulvar and/or perineal cyclical pain increasing during menstruations for 278 patients (98.2%). Five patients (1.8%) presented exclusively with other symptoms, e.g., painful bilateral polyps of the labia minora, cyclical bleeding, anal pruritus or infertility. Concurrent pelvic endometriosis occurred in 19 patients (6.1%). The median duration of symptoms before medical care was 12 months, ranging from 2 weeks to 20 years.

Out of the 281 patients for whom a clinical examination was described, 274 patients (97.5%) showed a vulvo-perineal nodule, mass or swelling, including one with vulvar ulceration, while 6 presented with bluish cutaneous lesions (2.1%) and one with bilateral polyps of the labia minora (0.4%).

The different workups conducted in the 283 patients are described in Table 4. The most common workup assessment was the serum level of cancer antigen 125 (CA125), measured in 105 patients (37.1%), followed by pelvic ultrasound (34.9%) and perineal ultrasound (27.%). Other workup examinations were performed in <10% of the cases. Slightly elevated serum levels of CA125 were found in 6.5–46.7% of patients with perineal endometriosis (10, 93, 94, 96). Pelvic ultrasound was mostly performed to exclude pelvic endometriosis. Perineal and endoanal ultrasound (50, 93, 94, 96), as well as magnetic resonance imaging (MRI) (94, 95) helped to describe precisely the size of the lesions and to diagnose and assess the extent of the anal sphincter involvement. Well-defined hypoechoic solid or cystic masses with hyperechoic spots or strands representing fibrosis within the scar tissue have been described (79, 97). Increased vascularity and spiculated borders with a single vessel entering the nodule from the periphery has also been observed (98). Fine needle aspiration cytology (FNAC) or biopsy of the lesions was reported in 19 patients (6.1%) and confirmed the diagnosis before surgical excision in 16 patients (84.2%) while it didn't confirm the presence of endometriosis in two patients [granulation tissue and old hemorrhage (22, 63)]. One biopsy result was not described (36).

**Table 4:** Work-up for vulvo-perineal endometriosis.

	**Number of patients (/283)**	**%**
Serum level of CA 125	105	37.1
Pelvic Ultrasound	99	34.9
Perineal ultrasound	78	27.6
MRI	19	6.7
Biopsy	17	6
CT-scan	9	3.2
Endoanal ultrasound	8	2.8
Sigmoidoscopy	7	2.5
Anal Manometry	4	1.4
FNAC	2	0.7
Dermoscopy	1	0.4

*CA 125, cancer Antigen 125; MRI, magnetic resonance imaging; CT-scan, Computerized Tomography scan; FNAC, fine needle aspiration cytology*.

All but one patients (282/283) underwent surgical excision of the perineal mass. In the patient where the perineal lesion was left, a total abdominal hysterectomy with bilateral salpingo-oophorectomy was performed to induce menopause and decrease pain-related symptoms (69). The detailed technique of the different surgical procedures was usually not described, except in two studies mentioning the following specifications i.e., narrow excision with deep surgical margins of 0.3–0.5 cm (10) or complete excision with a surgical margin of 0.5–1 cm (94). “Excision” or “surgical excision” without further details was mentioned for 148 patients (52.5%). Amongst the remainder, 73 had a “complete excision” (25.6%), 32 benefited from a “complete narrow excision” (11.3%) and 10 patients from a “wide excision” (3.5%). Eight surgeries were described as “incomplete” (2.8%). Histological findings confirmed endometriosis in all cases. Eighty-eight patients (28.1%) received hormonal therapy, either pre-or post-operatively for 3–6 months (Table 5).

**Table 5:** Hormonal therapy.

**Année**	**Number of patients**	**Age**	**Rx Pre-surgery**	**Time (month)**	**Traitement**	**Rx Post-surgery**	**Time**	**Follow-up (months)**	**Récurrence**
Shin et al. (67)	1	33	Danazol and GnRHa	2	Radical hysterectomy + bilateral oophorectomy with partial excision with cautherization of perineal lesions	local estrogens	NA	6	No
Liang et al. (2)	1	30	Danazol 800 mg	1	Excision	/	/	12	No
	1	33	Danazol irregularly	NA	Excision	/	/	12	No
Katz et al. (61)	1	14	Oral contraceptives	NA	Excision	/	/	4	No
Kang et al. (31)	1	32	GnRHa	3	Excision	/	/	NA	NA
Yogini et al. (35)	1	34	/	/	Drainage and excision	GnRHa	NA	NA	NA
Kahraman et al. (38)	1	19	/	/	Complete excision	Oral contraceptives	3	3	1
Zhu et al. (93)	13	30.67	GnRHa	3–6	Complete excision	GnRHa	3–6	9–168	No
	*7*		*GnRHa*	*3– 6*	*Incomplete excision*	*GnRHa*	*3–6*	*4–12*	*7*
	*1*		*Contraceptives 17alpha-hydroxyprogesterone caproate 250 mg im/28 days and tamoxifen 10 mg twice a day*	*15*	*Complete excision*	*Contraceptives 17alpha-hydroxyprogesterone caproate 250 mg im/28 days and tamoxifen 10 mg twice a day*	*8*	*84*	*1*
Hazard et al. (14)	1	15	/	/	Excision	Oral contraceptivew	NA	NA	No
Ngu et al. (63)	1	30	GnRha	3	Excision	/	/	NA	NA
Ruiz de Gauna et al. (20)	1	32	/	/	Wide excision	GnRHa	3	NA	No
Chen et al. (10)	10	33.4	GnRHa, Nemestran, DMPA	1– 6	Narrow excision	GnRHa, Nemestran, DMPA	1–6	NA	No
	1		GnRHa	3	Narrow excision	IUD-LNG	NA	NA	No
	*9*		*GnRHa*	*3–5*	*Narrow excision*	*/*	*/*	*12*	*1*
	2		/	/	Narrow excision	GnRHa	4	NA	No
	*1*		*DMPA*	*3–6*	*Incomplete excision*	*DPMA*	*3–6*	*72*	*1*
Hakimi et al. (80)	1	28	/	/	Excision (difficult)	LHRH	6	NA	NA
Grimstad et al. (24)	*1*	*29*	*/*	*/*	*Excision*	*Oral contraceptives*	*65*	*72*	*1*
Jeyaseelan et al. (83)	1	38	/	/	Excision	GnRHa	3	NA	NA
Li et al. (94)	*4*	*34.35*	*/*	*/*	*Surgical excision*	*GnRHa*	*3*	*12*	*1*
	2		/	/	Surgical excision	Mifepristone	1	NA	No
	1		/	/	Surgical excision	Progesterone	NA	NA	No
Sharp et al. (90)	1	39	/	/	Large biopsy	Dienogest 2 mg	6 months	NA	NA
Baba et al. (55)	1	35	/	/	Complete excision	GnRH	NA	NA	NA
Sharm et al. (89)	1	37	/	/	Wide excision	GnRHa	1	6	No
Saloum et al. (58)	1	34	/	/	Excision	GnRHa	3	NA	No
Wallace et al. (17)	1	42	/	/	Excision	Oral contraceptives	12	12	No
Liu et al. (96)	1	34	Marvelon	NA	Complete excision	GnRHa	3	85	No
	12	31.8	/	/	Complete excision	GnRHa	3–6	45.5	1 (12 months)
	1	37	Mifepristone	3	Complete excision	Mifepristone	3	75	No
	1	38	GnRHa	3	Complete excision	Mifepristone	3	62	No
	2	34.5	GnRHa	3	Complete excision	GnRH	3	46	No
	2	30.5	GnRHa	3 to 5	Complete excision	/	/	23.5	No
	88								

A follow-up period was mentioned in 61 studies, with a median value of 10 months (range from 1 to 108 months). Recurrent lesions have been reported in 29 patients (10.2%) and are presented in Table 6. Out of these patients with recurrence, 13 benefited from hormonal treatment pre-or post-operatively (44.8%) (nine GnRHa, three oral contraceptives, and one DMPA) while 16 didn't receive any additional treatment.

**Table 6:** Recurrences of perineal endometriosis after initial treatment.

	**Number of patients**	**ASI**	**Initial treatment**			**Follow-up (months)**
			**Surgical**	**Hormonal**	**Duration**	
Prince et al. (41)	1	No	Excision	No	NA	6
Trampuz et al. (76)	1	yes	Excision	No	NA	3
Swerdlow et al. (69)	1	No	TAH + BSO	No	NA	3
Gordon et al. (22)	1	yes	Excision	No	NA	5
Liang et al. (2)	1	NA	Excision	No	NA	12
Kahraman et al. (38)	1	No	CE	Oral contraceptives	NA	3
Nominato et al. (92)	1	NA	Excision	No	NA	NA
Eyvazzadeh et al. (19)	1	No	Biopsy	No	NA	60
Iqbal et al. (28)	1	No	Incision and drainage	No	NA	6
Zhu et al. (93)	7	Yes	IE	GnRHa pre or post op	3–6 months	4–12
	1	NA	CE	Postop contraceptive pills 17alpha-hydroxyprogesterone caproate 250 mg intramuscular injection per 28 days and tamoxifen 10 mg twice a day for 8 months	8 months	84
Chen et al. (10)	1	Yes	NE	GnRHa Pre-op	3 months	12
	1	Yes	IE	DMPA pre- and post-op	3–6 months	72
Jain et al. (32)	2	No	Excision	No	NA	3–7
Grimstad et al. (24)	1	No (clitoris)	Excision	Oral contraceptives	NA	72
Li et al. (94)	1	NA	CE	GnRH	3	12
Matalliotakis et al. (95)	2	NA	Excision	NA	NA	NA
Liu et al. (96)	3	No	CE	No	NA	12–36
	1	No	CE	GnRHa post-op	3 months	12
	29					

*IE, incomplete excision; CE, complete excision; NE, narrow excision; TAH + BSO, total abdominal hysterectomy + bilateral salpingo-oophorectomy; GnRHA, gonadotropin-releasing hormone analog; DMPA, Depot medoxyprogesterone acetate; NA, not available*.

## Discussion

While vulvo-perineal endometriosis presents mostly after perineal trauma, its exact etiology remains unclear, even if major progress has been achieved in the field over the last decades. Etiopathogenesis of endometriosis has generally been related to endometrial implantation, coelomic metaplasia, lymphatic dissemination and hematogenous spread (1) and the origin of extrapelvic endometriosis is not well-deciphered. As pelvic endometriosis can be considered as three separate entities (peritoneal, ovarian and recto-vaginal (deep) lesions) with different pathogeneses (99, 100), vulvo-perineal endometriosis could potentially also be separated between cystic and nodular lesions with distinct etiologies and treatment. Direct mechanical implantation seems to be the most plausible hypothesis for explaining scar endometriosis after obstetrical and gynecological procedures. According to this theory, mechanical dissemination during normal vaginal delivery, for example, allows transplantation of viable decidual endometrial cells into the episiotomy wound or perineal tear (2, 34, 53, 93, 101). We must note that scar endometriosis may as well-rarely be seen after a number of general surgical procedures like appendicectomy, inguinal hernial repair, laparoscopic cholecystectomy or even laparoscopic gastric by-pass (9). Nevertheless, perineal endometriosis has also been described in patients without any previous vulvo-vaginal trauma. In that respect, we found 13 cases of primary perineal endometriosis without vaginal birth, previous perineal injury in the literature. Different explanations of the pathogenesis of spontaneously developing perineal endometriotic lesions have been put forward with the most likely being lymphovascular dissemination (39, 53). However, the presence of endometriosis in the labia majora could also be explained by the direct spread of pelvic endometriosis along the round ligaments or Nuck canal's remnants while a solitary focus in the Bartholin's gland could theoretically be attributed to coelomic metaplasia (57). Eventually, other factors, such as immunological, genetic and familial factors, could be involved in the pathogenesis of this disease (94, 102).

Few studies have reported the incidence or prevalence of vulvo-perineal endometriosis. After vaginal delivery, the highest incidence rate of perineal endometriosis was reported by Nominato et al. representing 0.06% of patients, compared to 0.2% abdominal scar endometriosis after cesarean section (92), while perineal endometriosis only represents a proportion of 0.37% of women treated surgically for endometriosis (10).

Besides the rarity of vulvo-perineal endometriosis, its variability in clinical presentation makes this condition hardly recognized by some healthcare professionals leading to a delayed diagnosis. Misdiagnoses have also been reported such as herpes outbreak or perianal sepsis in the presence of vulvar pain with ulcerations or perianal swelling, respectively (14, 28). For instance, we showed a mean duration of symptoms of 12 months, ranging from 2 weeks to 20 years in our review meaning that half of them had to endure their pain for more than a year before being correctly diagnosed (94).

Early diagnosis and treatment are however recommended in order to prevent adverse complications such as long-term psychological distress, progressive involvement of the surrounding and adjacent tissues such as anal sphincter or rectum and potential malignant degeneration.

A detailed medical and surgical history associated with a thorough clinical exam are of great importance for accurate diagnosis. Good clinical vulvar and vaginal examination, including speculum and bimanual examination, are primordial to fully describe the perineal lesions. Rectal examination should be performed routinely in case of perineal lesions especially if suspicion of anal sphincter involvement (ASI). Clinically, endometriosis of the perineum and vulva presents as ill-defined papule or nodule, generally hard, usually located near a surgical scar, potentially skin-colored, dark-red, brown, or blue-black cystic (10, 103). It is mainly accompanied by cyclic pain and swelling, or periodic leakage of dark colored fluid during menses attributable to the fact that endometrial implants behave like normal endometrium. Some cases show neither discoloration of the perineal skin, nor local swelling nor intermittent leakage (32). Other symptoms can include dyspareunia. Interestingly, Zhu et al. described three criteria i.e., history of past perineal tear of episiotomy during vaginal delivery, presence of a tender nodule or mass at the perineal lesion on clinical exam and history of progressive and cyclic perineal pain which when all met provide a 100% positive predictive value (93). Concomitant pelvic endometriosis was found in 6.1% of patients in our systematic review. These results concord with the literature suggesting that scar endometriosis is not a risk factor for pelvic endometriosis (104, 105).

The differential diagnosis in such patients includes, but is not limited to, anal fistula, abscesses, suture granulomas, Bartholin cysts or bartholinitis, hernias, hematoma, sebaceous cyst, lipoma, herpes, neoplastic tissue or metastatic carcinoma, traumatic neuroma, desmoid tumor and anal melanoma (9, 28, 42, 45, 46). A perineal mass discovered in menopaused women should be considered as malignant until proven otherwise. Malignant degeneration occurs infrequently for cutaneous endometriosis representing 0.3–1% of endometriosis located in surgical scars (106). It is difficult to distinguish benign from malignant perineal endometriosis based on symptoms and clinical examination and a biopsy or surgical excision will always be necessary to confirm the diagnosis of malignant transformation (13). Histological observations are dominated by endometrioid carcinoma and sarcoma (107), but can also present as dermatosarcoma, clear cell carcinoma and serous papillary cystadenocarcinoma (87, 106, 108–116). As malignant transformation appears uncertain, unpredictable and may be very delayed, long-term follow-up is recommended.

The work-up appeared to be very variable depending on the medical team dealing with the patients. It was therefore difficult to evaluate the sensibility and specificity of each exam for perineal endometriosis as most of them have been realized in only a tiny proportion of the patients in this study. Levels of serum CA125 did not seem to be effective in diagnosing perineal endometriosis since it was usually normal or slightly increased. With regard to perineal ultrasonography, variable sonographic features were seen, as it is the case for abdominal wall endometriosis, which could make the diagnostic process more challenging but it remains useful to describe precisely the size of the lesions and to assess the extent of the ASI. Preoperative endoanal ultrasonography has also been described as a reliable technique for visualizing perianal endometriosis and diagnosing ASI, enabling the surgeon to determine the extent of an operative procedure and the possible need for a sphincteroplasty (46, 86). Ultrasonographic features of the lesion are usually similar to those observed with perineal ultrasonography with the advantage that it better reveals the involvement of the anal sphincter (10). Even if only 6.1% of the patients in this review benefited from this exam, magnetic resonance imaging (MRI) could become the modality of choice for perineal imaging (117, 118) as pelvic MRI has greater sensitivity (90–92%) and specificity (91–98%) for the diagnosis of endometriomas when compared to other non-invasive methods (119, 120). Vulvo-perineal localizations are easily identified on T1-weighted fat-suppressed images as hyper-intense spots within the perineum. Multilobular mass with inner hemorrhage, localized or diffuse vulvovaginal wall thickening, hemorrhagic or spiculated masses and distortion can also be observed (94, 117). MRI also helps in assessing the extent of the anal sphincter involvement (35). Depending on the availability in each center, we suggest that pelvic and perineal ultrasound as well as MRI should be performed in all patients and associated with endoanal ultrasound when there is a suspicion of ASI.

Histology is the hallmark of diagnosis which shows endometrial glands, stroma, and hemosiderin pigment. Generally, diagnosis is easy with a microscopic examination of a standard hematoxylin and eosin (H&E)-stained slide. Immunostaining for CD10 (neprilysin, a cell-surface metalloendopeptidase expressed in normal and ectopic endometrial stroma) increases the sensitivity compared to H&E staining (17, 19). Evidencing the estrogen receptor (ER) and progesteron receptor (PR) may help to identify endometrial glands (121). Furthermore, it is important to keep in mind that cutaneous endometriotic lesions show a broad spectrum of metaplastic changes and that all types of müllerian differentiation can be discovered (122) which make the diagnosis on FNAC or biopsy challenging for the unexperimented histologists.

Treatment of vulvo-perineal endometriosis includes usually surgical excision with or without hormonal suppression (GnRHa, oral contraceptives, progestins) (2, 10, 15, 123). It seems that complete excision should be the treatment of choice as it decreases the risk of recurrence (10, 93) and could reduce consequently the risk of malignant degeneration (109). Care must be applied to avoid rupturing the mass during surgery with its consecutive risk of re-implantation or leaving endometriotic remnants. To this end, excision of surrounding fibrous tissue has been suggested (94) although recommendations with respect to the surgical technique, e.g., surgical margins needed to decrease the risk of recurrence, are not available so far. Precise description of the surgical procedure including technique and margins is most often missing and awaited in future studies. When the anal sphincter is involved, complete narrow excision or wide excision of the endometrial tissue with a good healthy margin have been proposed with primary sphincteroplasty using the apposition or overlapping technique (10). Although symptomatic relief could be achieved with hormonal intervention, complete surgical excision still remains the best treatment for perineal endometriosis and often leads to permanent cure (71). As expected, large and deep lesions to the muscle or the fascia might be more difficult to excise completely. In large lesions, complete excision of the lesion may entail a synthetic mesh placement, tissue transfer for closure after resection (124) or combined surgery with gynecologic and plastic surgeons (125). It is however important to keep in mind that endometriosis remains a benign condition allowing conservative surgery and that decaying surgery is not recommended even in very large lesions. The type of resection should be based on the patient's age and desire for future pregnancy and the decision should be made only after possible outcomes of the different approaches have been discussed with the patient (86). Some authors have suggested that wide excision with primary sphincteroplasty could be optimal in younger patients, obviating the need for additional therapy, while narrow or incomplete excision with subsequent hormonal therapy could be advantageous in older patients closer to menopause to lessen the risk of incontinence due to sphincter resection (93). 28.1% of patients in this review received hormonal treatment pre-or post-operatively. As described in pelvic endometriosis, hormonal treatment could stabilize the size of cystic lesions and reduce pain as endometriosis is an estrogen-dependent process (126–129). Some authors suggested that massive lesions with anal sphincter involvement should be treated by hormonal therapy before surgery to reduce the size of the perineal mass (63). It should be noted that perineal endometriosis persists with medical treatment alone as it was always found on histology when hormonal treatment was followed by surgical excision. Various authors have reported the administration of a gonadotropin-releasing hormone analog to prevent recurrence (10, 31, 96). When complete wide excision, as reported by Zhu et al. (14), was performed, the recurrence rate was lower (3.3%) than the overall rate of 9.3% found in our review compiling all kinds of excisions, suggesting that recurrence was presumably due to incomplete removal of the lesions rather than to the absence of hormonal treatment (14). In addition, preoperative hormone therapy did not improve outcomes compared to surgery alone in patients with ASI (10). Results on hormonal therapy for abdominal wall or abdominal scar endometriosis are similar to those presented in this review showing a possible temporary relief of symptoms or potential slight reduction of the lesions' size easing the surgical resection but the bulk of evidence shows a low degree of efficacy. Currently, available data do not comment on best practices for the perioperative management of cutaneous endometriosis (104, 105, 130–134).

The limitations of this systematic review include the level of evidence due to the nature of the studies i.e., retrospective and case reports and the important variations in clinical management of vulvo-perineal endometriosis described in the available studies such as methods of diagnosis, surgery procedure's details and hormonal therapy. At present, there are no comparative studies to provide accurate and evidence-based guidelines regarding optimal diagnostic methods, treatment options and outcomes for endometriosis involving the perineum.

Although evidence-based guidelines cannot be retrieved from this systematic review due to the reasons mentioned above, Table 7 summarizes all the suggested recommendations based on our results and on the available literature. To improve our knowledge on this rare condition, we suggest developing a international database on vulvo-perineal endometriosis. Any future study regarding this type of endometriosis should include the data described in Table 8.

**Table 7:** Summary of recommendations about vulvo-perineal endometriosis.

**Early diagnosis and treatment** are recommended in order to prevent adverse complications.
**A detailed medical and surgical history** associated with a **thorough clinical exam** should be realized (vulvar and vaginal examination with rectal examination in case of suspicion of ASI).
**Pelvic and perineal ultrasound** as well as **pelvic MRI** should be performed in all patients and associated with endoanal ultrasound when there is a suspicion of ASI.
**Histology** is the hallmark of diagnosis.
**Complete excision** should be the treatment of choice as it decreases the risk of recurrence and could reduce consequently the risk of malignant degeneration.
**Hormonal treatment** could be proposed to attempt to decrease the size of a large lesion before surgery or to avoid recurrence of the lesion.
**Long-term follow-up** is recommended, as malignant transformation appears uncertain, unpredictable and may be very delayed.
**Every case of vulvo-perineal endometriosis should be reported** describing in details the previous history, the clinical management and the treatment received.

**Table 8:** Data to report in case of vulvo-perineal endometriosis.

Patient
Age
BMI
Ethnicity
Obstetrical History
Gravidity - Parity
Type of delivery
Episiotomy - Perineal laceration - Perineal tear (degree) - Perineal repair
Timing of any history
Medical or surgical history
Previous vulvo-perineal lesion, surgery or trauma
Previous abdominal surgery
Timing of any history
Symptoms
Beginning
Duration
Type (pain, localization, cyclical or not, …)
Latent period since trauma
Other symptoms
Clinical exam
Presence of a perineal mass or nodule
Size
Tenderness
Color of the Skin color
Detailed localization
Speculum examination
Rectal examination
Anal Sphincter Involvment
Work-up
Perineal ultrasound
Pelvic ultrasound
Pelvic MRI
Perianal ultrasound
Biopsy or FNAC
Other
Association with pelvic endometriosis
Treatment
Surgery
Excision or biopsy
Detailed procedure
Margins
Spillage
Type of closure
Type of repair in case of ASI
Hormonal
Pre-or post-surgery
Type
Duration
Comparison of symptoms and clinical exam before and after treatment
Histology
Follow-up
Recurrence
Type
Timing after treatment
Malignant transformation

In conclusion, vulvo-perineal endometriosis is a rare entity with ~300 cases reported in the literature since 1923. With the available knowledge shown in this systematic review, we encourage all practitioners to think about perineal endometriosis in case of perineal cyclical pain with or without previous perineal damage. Diagnosis should be done with clinical exam, perineal ultrasound and pelvic MRI when available. In case of anal sphincter involvement, perianal ultrasound should be performed. Surgical excision of the lesion should be realized in order to remove the lesion and to confirm the diagnosis histologically. Hormonal treatment could be proposed to attempt to decrease the size of a large lesion before surgery or to avoid recurrence of the lesion. As evidence-based approach to the diagnosis, treatment and recurrence rate of affected patients remains a challenge given its low prevalence, the variations in management found in the articles included and the limited quality of available studies, we suggest that a prospective database on vulvo-perineal endometriosis should be generated to increase knowledge but also awareness among healthcare professionals and optimize patients' care.

## Data Availability Statement

The raw data supporting the conclusions of this article will be made available by the authors, without undue reservation.

## Author Contributions

CM: search strategy, screening of studies, data extraction, manuscript writing, and final revision. ZC: screening of studies. J-LS, ML, and PJ: final revision. VT: screening of studies and language revision. CW: search strategy, manuscript writing, and final revision. All authors contributed to the article and approved the submitted version.

## Conflict of Interest

The authors declare that the research was conducted in the absence of any commercial or financial relationships that could be construed as a potential conflict of interest.
